# Cariprazine Use in Early Psychosis: Three Case Reports

**DOI:** 10.3389/fpsyt.2021.788281

**Published:** 2021-12-16

**Authors:** Ricardo Coentre, Rodrigo Saraiva, Carolina Sereijo, Pedro Levy

**Affiliations:** ^1^PROFIP–Programa de Intervenção nas Fases Iniciais da Psicose, Department of Psychiatry, Centro Hospitalar Universitário Lisboa Norte, Lisbon, Portugal; ^2^Faculdade de Medicina, Universidade de Lisboa, Lisbon, Portugal

**Keywords:** cariprazine, early intervention, first-episode psychosis, schizophrenia, early psychosis, case report

## Abstract

**Objective:** Cariprazine is a new atypical antipsychotic approved for the acute and maintenance treatment of schizophrenia (1, 2) and for the treatment of manic or mixed episodes associated with bipolar I disorder (1). Recently, cariprazine also got extended FDA-approval for the treatment of depressive episodes in adults with bipolar I disorder (3). The use of low doses of atypical antipsychotics is an essential component of early intervention in psychosis. For its particular performance and tolerability, cariprazine is becoming an important option for the treatment of first-episode psychosis.

**Method:** Three patients experiencing first-episode psychosis (FEP) were successfully treated with cariprazine. Two patients were in their first months of the disease, and the third patient was in his third year after the FEP.

**Results:** The three patients had a diagnosis of non-affective FEP, which includes schizophrenia, delusional disorder, and schizoaffective disorder. One of them was in their third year after the FEP with a predominance of negative symptoms at this stage of the disorder. All the patients were treated with cariprazine with a target dose of 3–4.5 mg/day. The three patients showed improvements in their psychosis, including a decrease in negative symptoms. No significant side effects were reported.

**Conclusion:** Our three case reports indicate that cariprazine is an atypical antipsychotic beneficial in the treatment of early psychosis. Treatment with low doses of cariprazine could be effective and tolerable in this phase of the disorder. Future studies with longer follow-up of FEP patients are recommended to confirm these positive results of cariprazine in the early phases of psychosis.

## Introduction

Psychotic disorders are severe mental illnesses that affect about 3% of the general population (4). Incidences of psychosis are highest in young, male, and ethnic minority patients (5–7). Also, patients with non-affective psychosis have higher incidence rates of psychosis compared to patients who experience affective psychosis (8). Psychosis has a significant impact on patients, families, and society. In Europe, expenses associated with psychotic disorders were over ninety-four billion euros in 2010, covering 5 million affected patients. The cost per patient per year was approximately €19,000 (9).

In previous decades, early intervention in psychosis has been adopted as a best practice by mental health specialists worldwide, as it has the most effective clinical and social results in the treatment of early psychosis, including first-episode psychosis (FEP) (10). Results of early intervention include reductions in the duration of untreated psychosis, hospitalization rates and duration of hospital stays, recurrence rate, and suicidal behavior. Patients saw improvement in quality of life, social functioning, and adherence to treatment (11). Early intervention in psychosis includes multidisciplinary teams utilizing several methods, including antipsychotic treatment and psychosocial interventions (12).

Low doses of atypical antipsychotics are an essential component of early intervention in FEP. Current guidelines indicate that antipsychotic medication should be administered with great care to individuals who are drug naïve (13–16). The classic approach “start low, go slow” is the fundamental attitude for dealing with antipsychotics in FEP. Antipsychotics should first be administered at a low dose, which is then increased depending on clinical results and the patient's tolerability, until reaching the patient's lowest effective dose. Atypical antipsychotics are chosen in FEP based on their side effects profile and comorbidity. Because of its negative metabolic profile, olanzapine and clozapine are indicated for second- and third-line treatment, respectively, in FEP (16).

As new oral atypical antipsychotics have become available over the past decade, monitoring clinical day-to-day experiences in various groups of psychotic patients is useful for clinicians. Cariprazine was approved for the acute and maintenance treatment of schizophrenia (1, 2) and for the acute treatment of manic or mixed episodes associated with bipolar I disorder (FDA, 2015). Recently, cariprazine also got extended FDA-approval for the treatment of major depressive episodes in adults with bipolar I disorder (3).

Cariprazine is similar to other atypical antipsychotics in exhibiting antagonistic activity at serotonin type 2A receptors (17). Cariprazine also acts as a partial agonist at the dopamine D3 and D2 receptors with high binding affinity and at the serotonin 5-HT1A receptor. Cariprazine has a similar profile to aripiprazole, except for D3, which has tenfold greater affinity than for D2, so high that extremely small doses are sufficient to get maximal D3 occupancy (18). This particular D3 receptor blockage could theoretically have pro-cognitive effects, antidepressant effects, and reduce the negative symptoms of schizophrenia (19). Cariprazine has other receptor properties, including moderate histamine antagonism, low α-1a antagonism, and no significant affinity for muscarinic cholinergic receptors (20).

Cariprazine metabolites, desmethyl-cariprazine and didesmethyl-cariprazine, have pharmacological properties similar to their parental drug, but the half-life of didesmethyl-cariprazine is considerably longer (1–3 weeks). Exposure to didesmethyl-cariprazine is several times higher than that for cariprazine. This long half-life of didesmethyl-cariprazine is important because it allows the development of a once-a-day oral formulation, which improves medication adherence. A missed dose of cariprazine may be associated with a lower risk of sub-optimal receptor binding compared to a drug with a shorter half-life. However, the longer half-life may also imply a prolonged duration of hypothetical adverse events after discontinuing the treatment (20).

Cariprazine's clinical profile is described in Table 1.

**Table 1:** Cariprazine clinical profile.

	**Target dose**	**Common side effects**	**Pharmacokinetics**	**Pharmacodynamics**	
				**Receptor subtypes**	**Activity**
• Acute and maintenance treatment of schizophrenia (FDA, EMA)	• 1.5–6 mg/day	• Extrapyramidal symptoms •Insomnia • Akathisia	• *Route of administration*: Oral • *Bioavailability*: protein binding ~96%; half-life ~1 week for the combined drug	*Dopamine*	
				• D_2S_ • D_2L_ • D_3_	Partial agonist Partial agonist Partial agonist
• Acute treatment of manic or mixed episodes associated with bipolar I disorder in adults (FDA)	• 3–6 mg/day		• *Metabolism*: hydroxylation and demethylation (via CYP3A4 CYP2D6) • *Elimination*: urine (21% of dose)	*Serotonin*	
				• 5-HT_1A_ • 5-HT_2A_	Partial agonist Antagonist
• Depressive episodes in adults with bipolar I disorder (FDA)	• 1.5–3 mg/day		• *Metabolism*: hydroxylation and demethylation (via CYP3A4 CYP2D6) • *Elimination*: urine (21% of dose) • *Main metabolites:* • Desmethyl-cariprazine and Didesmethyl-cariprazine	• 5-HT_2B_ • 5-HT_2C_ • 5-HT_7_ *Histamine* • H_1_ *Adrenergic*	Antagonist Antagonist Antagonist
					Antagonist
				• α_1_	Antagonist

*FDA, U.S. Food and Drug Administration; EMA, European Medicines Agency; D, dopamine; 5-HT, serotonin; H, histamine; α, alpha*.

The clinical use of cariprazine was studied in three patients with early psychosis. The data is still scarce, but as more patients use cariprazine during the first years of psychosis, the amount of data continues to increase. These case reports could improve the understanding of the use of the recent antipsychotic cariprazine in the early stages of psychosis.

## Case Reports

### Case 1

Case 1 focuses on a 26-year-old male university student with a history of 8 weeks of clinical picture, characterized by disorganized thoughts and behavior, hypochondriac delusions, reference delusions, and insomnia. This clinical picture progressively worsened. He reported no history of medical comorbidities and had not previously experienced an acute psychiatric episode. He had a family history of alcohol use disorder from both parents. The patient had a history of cannabis use from when he was 19 years old. Initially, he used cannabis once a week, and within the last two years, this increased to daily use. He presented with the described psychotic symptoms to the emergency department of a university hospital in Lisbon, Portugal, where he was seen by a psychiatrist. He voluntarily stayed in the ward of the Department of Psychiatry with the diagnosis of non-affective FEP. During his stay in the ward, several tests were performed on him, including blood analysis with thyroid function, hepatitis B and C, syphilis and HIV serologies, vitamin B12 dosing, prolactin and cortisol levels, metabolic profile, and urine drug screening (cannabis, amphetamines, cocaine, and heroin). The results produced no abnormalities except for the urine screening for cannabis being positive. A cranial MRI, electrocardiogram, and electroencephalogram were also performed, and the results were in normal ranges. The patient was treated with cariprazine 1.5 mg/day during days 1 and 2, and afterwards titrated to 3 mg/day. Within the first 4 days, he took flurazepam 15 mg/day at bedtime, which was then tapered off. After 12 days, the patient showed significant improvement in disorganized thoughts and delusions, and his insomnia ameliorated after 2 days of being in the ward. He had no side effects with psychopharmacology. The patient was discharged while maintaining cariprazine 3 mg/day, which he has been adherent to. The total score of the Positive and Negative Syndrome Scale (PANSS) dropped from 78 (admission) to 41 (hospital discharge). The patient is currently stable on this treatment regimen with a follow-up of approximately 5 months and with good adherence to pharmacological treatment. Besides psychiatric follow-up, the patient has also been followed-up by a psychologist doing individual psychoeducation for psychosis. He did not use cannabis after discharge from the hospital. He returned to his studies at the university, and he is living with his girlfriend.

### Case 2

This is an unemployed 28-year-old man with a history of non-affective FEP, diagnosed three years prior. His previous psychosis was characterized by persecutory and reference delusions and auditory hallucinations. Prior to FEP, he had about one year of social withdrawal and a sudden interest in philosophy and psychology. The patient had a history of cannabis use since he was 21 years old. He had no family history of psychiatric disorders. He also had no significant medical history. During the FEP, the patient was hospitalized in the Department of Psychiatry for about 4 weeks. Several exams were performed consisting of blood analysis (including thyroid function, hepatitis B and C, syphilis and HIV serologies, vitamin B12 dosing, prolactin and cortisol levels), metabolic profile and urine drug screening, cranial MRI, electrocardiogram, and electroencephalogram. The test results produced no significant changes except for the cannabis screening being positive. During his stay in the hospital, he was medicated with aripiprazole 15 mg/day, which produced signs of improvement. Following his discharge from the hospital, he experienced about two years of negative symptoms with exuberant blunting affect and social withdrawal and no other symptoms or relapses. The patient continued to use cannabis, however, less frequently. At this time, the antipsychotic medication was switched to paliperidone 6 mg/day trying to improve these significative negative symptoms. This change in antipsychotic treatment produced no significant repercussions on the clinical picture of negative symptoms of the patient. After 6 months of treatment with paliperidone, the psychopharmacological treatment was again switched to cariprazine. Cariprazine was titrated to 4.5 mg/day (1.5 mg/day on days 1 and 2, 3.0 mg/day on days 4 and 5, and 4.5 mg/day after day 5). After 1 week, he started to feel better, saying, “It feels like I am not on medication.” The patient continued improving from the described negative symptoms over the following 6 months. He is now at about 3 years of follow-up after FEP, complying with medication and with no side effects. The PANSS negative subscale score diminished from 38 before starting treatment with cariprazine to 12 at 6 months of treatment. The patient is still using cannabis with a sporadic frequency (approximately once every six months). He started a new relationship and is working at a shop.

### Case 3

This patient is a 32-year-old female. She was an old-age caregiver with a history of about 12 weeks of a clinical picture characterized by persecutory delusions and auditory hallucinations. These psychotic symptoms were accompanied by psychotic anguish and emotional lability. This clinical picture started shortly after her divorce. The symptoms worsened, and the patient refused to leave her home because she felt insecure outside of it. Her sister, understanding the declining mental health of the patient, convinced her to go to a hospital. The patient was seen in the emergency department by a psychiatrist who advised her to stay in the hospital. She accepted, understanding it was a safe place for her. The patient had a history of hypercholesterolemia and had been medicated with simvastatin 20 mg/day. No other medical diseases were known, though the mother of the patient had been diagnosed with schizophrenia. Thus, she was hospitalized in the Department of Psychiatry with non-affective FEP. During her stay in the hospital, several exams were performed on her consisting of a complete blood analysis (including thyroid function, hepatitis B and C, syphilis and HIV serologies, vitamin B12 dosing, prolactin and cortisol levels and urine drug screening), cranial CT, and electrocardiogram. None of the exams showed significant abnormalities. The patient's metabolic profile revealed that her dyslipidemia was controlled with statin treatment (total cholesterol: 176 mg/dL; LDL: 97 mg/dL; HDL: 55 mg/dL; triglycerides: 142 mg/dL). During the hospital stay, the patient was medicated with cariprazine 1.5 mg/day that was then titrated to 3.0 mg/day (1.5 mg/day on days 1 and 2, 3.0 mg/day afterward). In the first three days of treatment, the patient complained of sleepiness during the day, which was overcome by changing the timing of her cariprazine dosage (1.5–3.0 mg/day) from breakfast to bedtime. She experienced a progressive remission of the described psychotic clinical picture. The patient was discharged after spending 15 days in the hospital. The PANSS total score reduced from 74 (admission) to 34 (hospital discharge). The patient had been followed-up in a psychiatric outpatient clinic at a secondary hospital. After 12 months, she showed improvement with complete remission of psychotic symptoms with no significant side effects and adhering to antipsychotic treatment. She also returned to her job.

## Discussion

The three case reports seem to demonstrate the efficacy of cariprazine in early psychosis, which is new, although expected. In all three patients, there was an improvement in the clinical picture of psychosis. Patients 1 and 3 experienced a remission of psychotic positive symptoms, and patient 2 experienced improvement of negative symptoms while maintaining remission of positive symptoms. The recovery of the three patients was positive, not only with the remission of positive and negative symptoms, but also with the return of functioning.

The results were similar to other trials that demonstrated the efficacy of cariprazine in acute schizophrenia (21–24). In addition, reviews and meta-analysis proved the efficacy of cariprazine in acute schizophrenia (25–27). These studies showed the superiority of administering cariprazine vs. placebo, showing an improvement of the PANSS total score, positive and negative subscale scores, and of the Clinical Global Impression score. For example, a phase III study evaluated the efficacy and safety of cariprazine in acute exacerbation of schizophrenia (23). This was a 6-week double-blind trial, where patients were randomized to three groups: placebo, cariprazine 3 to 6 mg/d, or cariprazine 6 to 9 mg/d. The primary outcome was a change from baseline to week six in the PANSS score. Common adverse effects in the cariprazine groups included mild to moderate akathisia, extrapyramidal disorder, and tremor, with no or very low changes in metabolic measures and decrease of prolactin in all groups. These results show that cariprazine is effective and well tolerated. A recent published systematic review and meta-analysis compared the efficacy and tolerability of 32 oral antipsychotics for the acute treatment of adults with multi-episode schizophrenia (28). The results must take into consideration the relatively small number of participants assigned to the treatment with cariprazine and the existence of few trials with direct comparison with cariprazine, almost all with placebo. The results show very low changes in positive and negative symptoms and moderate changes in depressive symptoms. For all causes of discontinuation, the results also show that cariprazine has a low rate compared with placebo.

Cariprazine was also approved for maintenance treatment in schizophrenia. A multinational, randomized, double-blind, and placebo-controlled study evaluated the role of cariprazine for relapse prevention in adults with schizophrenia (29). Stable patients who completed open-label treatment with cariprazine 3–9 mg/day were placed in randomized groups to continue taking cariprazine (3, 6, or 9 mg/day) or a placebo for double-blind treatment for up to 72 weeks. Long-term cariprazine treatment was significantly more effective than the placebo for relapse prevention in patients with schizophrenia. Interestingly, there are reports of using cariprazine as an add-on treatment for treatment-resistant schizophrenia with partial response to clozapine (30). In this case series, cariprazine add-on to clozapine showed excellent efficacy and rapid effect in the treatment of patients with a partial response to clozapine. The tolerability of this association was excellent without reported significant adverse effects.

Cariprazine is one of the newest antipsychotics available to treat FEP. The results presented from the three case reports were positive when using cariprazine in early psychosis. Besides its efficacy in treating positive psychotic symptoms, the case studies showed that cariprazine produced good efficacy on negative symptoms, as with patient 2. Therefore, negative cluster symptoms are important actions of cariprazine (31–34). Pre-clinical studies have proven that cariprazine is effective in the treatment of anhedonia, depressive, and anxiety behaviors (35, 36). Clinical trials also showed efficacy on negative symptoms in patients with schizophrenia. Post-hoc analysis of data from acute schizophrenic patients, with a high score of negative symptoms and a low or moderate score of positive symptoms on the PANSS scale, reported improvement in the PANSS factor score for negative symptoms with cariprazine vs. placebo (37). A randomized, double-blind trial comparing the efficacy of risperidone (4 mg/day) and cariprazine (4.5 mg/day) showed that cariprazine helped to reduce the PANSS factor score for negative symptoms from the baseline to week 26 compared to risperidone (32). This result supported the increased efficacy on negative symptoms while taking cariprazine.

The three case reports showed positive tolerability and safety of cariprazine. Even in FEP patients who usually are sensitive to antipsychotic side effects, cariprazine seemed to be well tolerated (38). In the three cases, no significant side effects were reported. Only one patient reported sleepiness during the day in the first days of cariprazine, which was easily resolved by changing its delivery to the end of the day. Other studies show that the overall possibility of adverse effects is similar for cariprazine and placebo at doses of 1.5–3 mg/day, while higher for doses of 4.5–6 mg/day (39). A higher risk of EPS-related symptoms (akathisia, tremor, and restlessness) and a slight increase in overall body weight can be seen with cariprazine (25, 40). A recently published paper made a pooled analysis of eight phase II/III studies to analyze the safety profile of cariprazine. It includes four short-term (6-week) and four long-term (≥6 months) studies that used the recommended 1.5–6 mg/d dose range for schizophrenia (41). The results showed that cariprazine was safe and well tolerated in the recommended dose. Mild and moderate akathisia was the most common adverse effect seen, but it resulted in few discontinuations. None of these adverse effects were seen in our patients. There are no significant impacts on the prolactin level, metabolic parameters, QT interval, and cardiovascular health (39, 40, 42). Cariprazine also showed a low chance of causing sleepiness and drowsiness, which is vital in terms of compliance with antipsychotic medication, namely in young people (40, 42, 43). Due to its low affinity to cholinergic receptors, there is no impairment in colon transit with cariprazine (42).

Cariprazine also seems useful in other clinically relevant situations frequently associated with schizophrenia. This is the case of obsessive-compulsive symptoms, frequently seen associated with schizophrenia in different phases of the disorder. There are published case reports where the add-on treatment with low-dosage cariprazine showed remission of these symptoms (44).

Some limitations exist regarding these three case reports, most notably some heterogeneities. First, we present here three different case reports regrading the time of cariprazine use: two reports initiated cariprazine immediately in the acute phase of FEP (cases 1 and 3) and the other one after three years of the FEP for the treatment of exuberant negative symptoms (case 2). Second, the follow-up of the three cases varies between the three case reports. The follow-up times are 5, 36, and 12 months, respectively, for case reports 1, 2, and 3. However, these differences between the three case reports presented also improve the knowledge and experience of the clinical use of cariprazine in different time phases of early psychosis.

## Conclusion

These case reports seem to indicate that cariprazine is effective and well tolerated in patients experiencing early phases of psychosis, namely those with non-affective FEP. Cariprazine seems particularly useful in patients where negative symptoms are evident. The safety and positive tolerability profile of cariprazine made it especially useful in young and active patients. Future studies should be performed to confirm the long-term positive results of using cariprazine in this type of patient.

## Data Availability Statement

The datasets used and/or analyzed during the current study are available from the corresponding author upon reasonable request.

## Ethics Statement

Ethical review and approval were not required for the study on human participants in accordance with the local legislation and institutional requirements. Written informed consent to participate in this study was provided by the participants.

## Author Contributions

RC, RS, CS, and PL collaborated on the clinical work. RC, RS, and CS completed the literature review. PL oversaw the process and contributed to content and editing. All authors contributed to manuscript revision and read and approved the submitted version.

## Funding

The open access fee for publication of these case reports is funded by Recordati.

## Conflict of Interest

The authors declare that the research was conducted in the absence of any commercial or financial relationships that could be construed as a potential conflict of interest.

## Publisher's Note

All claims expressed in this article are solely those of the authors and do not necessarily represent those of their affiliated organizations, or those of the publisher, the editors and the reviewers. Any product that may be evaluated in this article, or claim that may be made by its manufacturer, is not guaranteed or endorsed by the publisher.
